# Tendinosis develops from age‐ and oxygen tension‐dependent modulation of Rac1 activity

**DOI:** 10.1111/acel.12934

**Published:** 2019-04-02

**Authors:** Rowena McBeath, Richard W. Edwards, Brian J. O’Hara, Mitchell G. Maltenfort, Susan M. Parks, Andrzej Steplewski, A. Lee Osterman, Irving M. Shapiro

**Affiliations:** ^1^ Department of Orthopaedic Surgery Thomas Jefferson University Philadelphia Pennsylvania; ^2^ Division of Orthopaedic Research, Department of Orthopaedic Surgery Thomas Jefferson University Philadelphia Pennsylvania; ^3^ Philadelphia Hand to Shoulder Center Philadelphia Pennsylvania; ^4^ Department of Pathology, Anatomy and Cell Biology Thomas Jefferson University Hospital Philadelphia Pennsylvania; ^5^ The Applied Clinical Research Center, Department of Biomedical and Health Informatics Children's Hospital of Philadelphia Philadelphia Pennsylvania; ^6^ Division of Geriatric Medicine & Palliative Care, Department of Family & Community Medicine Thomas Jefferson University Philadelphia Pennsylvania

**Keywords:** fibrochondrocyte, hypoxia, Rac1, RhoA, tendinosis, tenocyte

## Abstract

Age‐related tendon degeneration (tendinosis) is characterized by a phenotypic change in which tenocytes display characteristics of fibrochondrocytes and mineralized fibrochondrocytes. As tendon degeneration has been noted in vivo in areas of decreased tendon vascularity, we hypothesized that hypoxia is responsible for the development of the tendinosis phenotype, and that these effects are more pronounced in aged tenocytes. Hypoxic (1% O_2_) culture of aged, tendinotic, and young human tenocytes resulted in a mineralized fibrochondrocyte phenotype in aged tenocytes, and a fibrochondrocyte phenotype in young and tendinotic tenocytes. Investigation of the molecular mechanism responsible for this phenotype change revealed that the fibrochondrocyte phenotype in aged tenocytes occurs with decreased Rac1 activity in response to hypoxia. In young hypoxic tenocytes, however, the fibrochondrocyte phenotype occurs with concomitant decreased Rac1 activity coupled with increased RhoA activity. Using pharmacologic and adenoviral manipulation, we confirmed that these hypoxic effects on the tenocyte phenotype are linked directly to the activity of RhoA/Rac1 GTPase in in vitro human cell culture and tendon explants. These results demonstrate that hypoxia drives tenocyte phenotypic changes, and provide a molecular insight into the development of human tendinosis that occurs with aging.

## 1 INTRODUCTION

Tendinosis—degenerative tendon injury—predominantly affects the elderly and results in significant medical costs and disability. Tendinosis is detected histologically by altered cellularity, vasculature, and changes in the tenocyte phenotype (Maffulli, Wong, & Almekinders, 2003). While it is well known that tendon vascularity decreases with age, the molecular etiology for tendinosis development remains unclear. Normally, tendons are composed of tenocytes, elongated fibroblasts located in tendon (Kannus, 2000), that are derived from mesenchymal stem cells (Maharam et al., 2015). Cells in diseased tendon, however, commonly exhibit a fibrocartilaginous phenotype and display a hyaline, mucoid, calcified, or fibrous appearance (Kannus & Jozsa, 1991). Interestingly, these same histologic changes noted in tendinosis tissue of elderly patients have also been noted to a lesser extent in tendons of asymptomatic aged individuals, suggesting that tendinosis results from sustained cellular responses to cues that exist physiologically in the aging tenocyte microenvironment.

One explanation for the altered cell phenotype observed in aged tendon is that human tenocytes develop a fibrochondrocyte phenotype depending on their microenvironment, in particular to changes in oxygen level effected by tendon vascularity. Tendon vascularity decreases with age (Brewer, 1979) and tendinosis increases in hypovascular areas of tendon (Millar et al., 2012). In vitro, the environmental oxygen tension has previously been considered as key to cell survival, while other studies have linked the importance of oxygen tension to maintenance of the tenocyte phenotype (Fehrer et al., 2007; Zhang & Wang, 2018). We hypothesize that the decreased vascularity of tendon that occurs with aging affects tenocyte metabolic and other intracellular signaling pathways, thus influencing the cellular phenotype.

Another explanation for the development of the tendinosis phenotype concerns the connective tissue mechanical environment, namely force transmission. Tendinosis has long been associated with repetitive use and mechanical overload as well as age (Yamaguchi et al., 2006). Studies of human tissues as well as animal models demonstrate a fibrochondrocyte phenotype—tenocyte rounding and collagen II secretion—in tendons exposed to an increased compressive mechanical force (Benjamin, Qin, & Ralphs, 1995). Signaling cascades involved in force transmission include the Rho/Rac GTPases. Rac1 is activated by tensile stretch, while RhoA is activated by compression (Haudenschild, Nguyen, Chen, D'Lima, & Lotz, 2008; Katsumi et al., 2002). Rho/Rac GTPases are not exclusively force‐activated: Rac1 and RhoA are also activated by hypoxia (Hirota & Semenza, 2001; Turcotte, Desrosiers, & Beliveau, 2003). It has previously been shown at the cellular level that force transmission through the RhoA/Rac1 GTPases is critical to other connective tissue cell phenotypes (McBeath, Pirone, Nelson, Bhadriraju, & Chen, 2004; Gao, McBeath, & Chen, 2010; Woods, Wang, Dupuis, Shao, & Beier, 2007). We hypothesize here that the change in tenocyte phenotype in aging tendon is due to altered RhoA/Rac1 signaling in a hypoxic microenvironment.

To test the hypothesis that the tendinosis phenotype is dependent on oxygen tension and mediated by changes in RhoA/Rac1 GTPase activity, we examined collagen production and calcification in aged tendinotic and young asymptomatic tendon tissue via Fourier‐transform infrared spectroscopy (FTIR). Aged diseased tendon tissue displayed decreased mature collagen and increased calcification as compared to young asymptomatic tendon tissue. Similarly, when aged tenocytes were cultured in vitro, we found that aged tenocytes calcified in hypoxia but not normoxia. Furthermore, hypoxia resulted in a fibrochondrocyte phenotype in all cell types in a density‐dependent manner. Investigation of the signaling mechanism during this transition revealed the fibrochondrocyte phenotype in aged tenocytes occurred with decreased Rac1 activity. In young hypoxic tenocytes, the fibrochondrocyte phenotype occurred with concomitantly decreased Rac1 activity coupled with increased RhoA activity. We confirmed that this hypoxic effect on the human tenocyte phenotype was linked directly to the activity of RhoA/Rac1 GTPase via pharmacologic and adenoviral manipulation in in vitro human cell culture and tendon explants. These results demonstrate that hypoxia drives transition of the human tenocyte to a fibrochondrocyte phenotype, and provide a molecular insight into the development of human tendinosis.

## 2 RESULTS

### 2.1 Aged and diseased tendon display altered cellular morphology and extracellular matrix production in vivo

**Figure 1:**
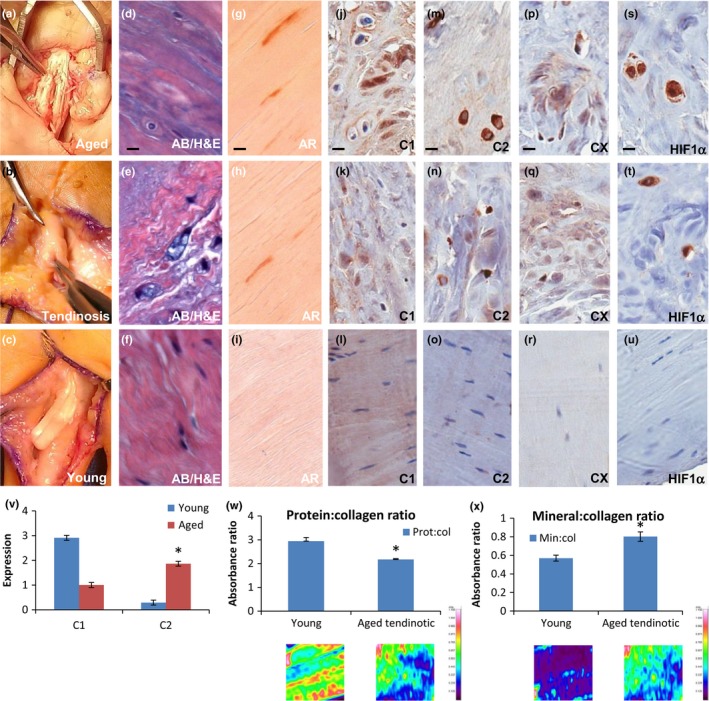
Aged and diseased tendon display altered cellular morphology and extracellular matrix production in vivo. (a–c) Intra‐operative photographs of (a) 84‐year‐old female patient undergoing fibrocartilage pulley release for recalcitrant flexor tendinosis (aged), (b) 42‐year‐old female patient undergoing tendinosis excision prior to reconstruction (tendinosis), (c) 30‐year‐old female patient undergoing primary tendon repair after acute laceration (young). Forcep tip delineates the flexor tendon. (d–f) Alcian blue (AB) histopathologic staining of (a‐c), respectively. Bar = 20 μm. (g–i) Alizarin red (AR) histopathologic staining of (a‐c), respectively. Bar = 20 μm. (j–u) Immunohistochemical staining of aged (j,m,p,s), tendinosis (k,n,q,t), and young (l,o,r,u) patient samples as in (a–c) for: col1α2 (j‐l), col2α1 (m–o), colX (p–r) and HIF‐1α (s–u). Bar = 20 μm. (v) Quantitation of immunoblot of collagen 1α2 and collagen 2α1 expression from young asymptomatic tendon (Young) or aged diseased tendon (Aged). *p < 0.05, *n* = 4. (w, x) FTIR of young asymptomatic tendon (Young) or aged diseased tendon (Aged tendinotic). (w) Protein:collagen ratio. (x) Mineral:collagen ratio. Spectrophotometric absorbance as shown below quantitation with absorbance scale to the right. *p < 0.05, *n* = 4

In surgical management of tendinosis, we have often observed tendon flattening and fraying in older affected individuals (Figure 1a), a thickened, bulbous appearance in younger affected individuals (Figure 1b) as compared to a striated, glassy gross tendon phenotype in young asymptomatic individuals (Figure 1c). Histologic evaluation reveals changes in the tenocyte phenotype in diseased tendon. In aged and young diseased tendon, tenocytes are round and surrounded by increased matrix metachromasia (Figure 1d,e), with areas of calcification (Figure 1g,h). Immunohistochemical staining of these areas reveals expression of collagen 1α2 (Figure 1j,k) but also fibrochondrocyte marker collagen 2α1 (Figure 1m,n), and mineralized fibrochondrocyte marker collagen X (Figure 1p,q). Interestingly, these areas correlate with hypoxia‐inducible factor 1α (HIF‐1α) expression, the highest levels of which are noted in the diseased tendon midsubstance, co‐localizing with the fibrochondrocyte and mineralized fibrochondrocyte phenotype (Figure 1s,t). In contrast, young asymptomatic tenocyte nuclei are flat, surrounded by streamlined collagenous matrix and no calcification (Figure 1f,i), express collagen 1α2 (Figure 1l) but not collagen 2α1, collagen X, or HIF‐1α (Figure 1o,r,u). Thus, in aged and young diseased tendon, we have noted a fibrocartilage and mineralized fibrocartilage phenotype in areas of tendon compression and fraying, correlating with HIF‐1α expression.

We subsequently investigated the molecular nature of the gross phenotypic changes observed in diseased tendon via immunoblot and FTIR. While asymptomatic tendon is composed almost exclusively of collagen I, aged diseased tendon demonstrated decreased collagen I and increased collagen II production by immunoblot quantitation (Figure 1v). Similarly, FTIR of aged diseased tendon revealed decreased mature collagen production and increased mineralization in comparison with young asymptomatic tendon (Figure 1w,x). We concluded based on these observations that human tendinosis in vivo is characterized by the development of a fibrochondrocyte and mineralized fibrochondrocyte phenotype, as demonstrated by gross morphology, histopathology, immunohistochemistry, protein, and molecular studies.

### 2.2 Aged tenocytes display altered cellular morphology and extracellular matrix production in vitro

**Figure 2:**
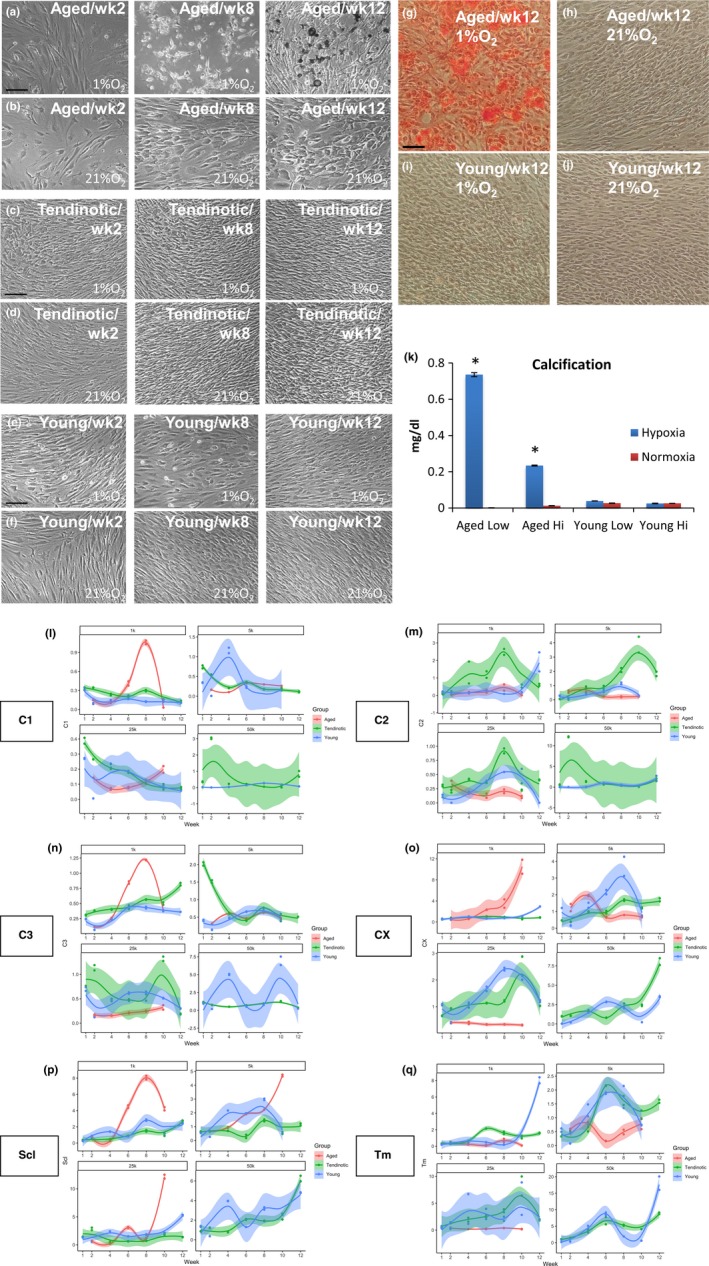
Aged tenocytes display altered cellular morphology and extracellular matrix production in vitro. Human tenocytes from aged (Aged), young tendinotic (Tendinotic), and young asymptomatic (Young) patients were cultured at low (1k/cm^2^ aged a‐b; 5k/cm^2^ young tendinotic c, d and young e, f) cell densities in hypoxic (1% O_2_; a, c, e) or normoxic conditions (21% O_2_; b, d, f) over twelve weeks. Shown are phase contrast micrographs of representative cells from week 2 (wk2), week 8 (wk8), and week 12 (wk12). Bar = 50 μm. (g–j) Brightfield photomicrographs of alizarin red stained aged (g, h) or young asymptomatic (i, j) tenocytes in hypoxic (1% O_2_, g, i) or normoxic (21% O_2_, h, j) conditions at week 12. (k) Spectrophotometric quantitation of calcification (mg/dL) as detected by alizarin red staining of aged (Aged) or young asymptomatic (Young) tenocytes cultured in hypoxic (Hypoxia, 1% O_2_) or normoxic (Normoxia, 21% O_2_) conditions at low (Low, 1k/cm^2^ for aged, 5k/cm^2^ for young asymptomatic) or high (High, 25k/cm^2^ for aged, 50k/cm^2^ for young asymptomatic) cell densities and harvested at week 12 in culture. *p < 0.05, *n* = 4. (l–q) qRT–PCR expression of aged (red), young tendinotic (green), or young asymptomatic (blue) tenocyte (C1, Scl, Tm, C3), fibrochondrocyte (C2), and mineralized fibrochondrocyte (CX) markers over a 12‐week timecourse, hypoxic (1% O_2_) as compared to normoxic (21% O_2_) conditions, at cell densities: 1k = 1 × 10^3^cells/cm^2^; 5k = 5 × 10^3 ^cells/cm^2^; 25k = 25 × 10^3 ^cells/cm^2^; 50k = 50 × 10^3^cells/cm^2^ (not available for aged tenocytes). C1 = col1α2; C2 = col2α1; C3 = col3; CX = colX; Scl = scleraxis; Tm = tenomodulin

Given the changes in tenocyte morphology and matrix production we observed in aged diseased tendon in vivo, we asked whether these changes exist in in vitro human tenocyte culture and if so, what factors are responsible for initiation of the altered phenotype. First, we isolated tenocytes from young patients undergoing debridement for tendinosis (<45 years, “Tendinotic”), as well as from asymptomatic young (<45 years, “Young”) and aged (>65 years, “Aged”) patients undergoing revision amputation for irreparable hand injury according to IRB‐approved and established protocols (Yao, Bestwick, Bestwick, Maffulli, & Aspden, 2006; Supporting Information Data S1). As tendinosis is noted in areas of altered vascularity and cellularity, we investigated the impact of environmental conditions—oxygen tension and cell density—on the tenocyte phenotype over time via qRT–PCR of extracellular matrix proteins found predominantly in tendon (collagen Iα2, collagen III, tenomodulin), as well as fibrocartilage (collagen IIα1), and mineralized fibrocartilage (collagen X) (Figure 2).

Cell shape varied between aged, young tendinotic, and asymptomatic young cells cultured in hypoxic (1% O_2_) versus normoxic (21% O_2_) conditions (Figure 2a–f). Aged tenocytes were consistently cuboidal, while young tendinotic and asymptomatic young tenocytes more elongated. Hypoxia resulted in cell rounding in both aged and young tenocytes. Interestingly, at low cell density in aged hypoxic tenocytes, we observed the development of prominent extracellular calcification by week 8, staining positively with Alizarin Red at week 12 (Figure 2g,k) in a density‐dependent manner (Figure 2k). In contrast, there was no mineral deposition by young hypoxic tenocytes, or aged or young normoxic tenocytes (Figure 2h,i,j).

Given the effect of cellularity on matrix mineralization in aged tenocytes, we then asked how hypoxia and cell density affected the expression of other extracellular matrix proteins. qRT–PCR of aged, young tendinotic, and young asymptomatic tenocytes in hypoxic compared to normoxic conditions revealed that hypoxia decreased collagen 1α2 expression in all cell types (Figure 2l); however, hypoxia increased collagen IIα1 expression in young tendinotic and asymptomatic young tenocytes (Figure 2m) and increased collagen X expression in all cell types (Figure 2o). The effect of hypoxia was density‐dependent, with greatest increases in collagen II in young tendinotic cells at high densities, and collagen X in aged tenocytes at low cell density (Figure 2m,o). Statistical analysis using Spearman's rho revealed significant correlations between collagen II and tenomodulin expression in young asymptomatic tenocytes (*p* < 0.001); collagen II, collagen X, and tenomodulin expression in young tendinotic tenocytes (*p* < 0.001); and collagen II and collagen X expression in aged tenocytes (*p* < 0.001) (Supporting Information Data S2).

As tendon tissue contains mature tenocytes as well as a small percentage (1%–3%) of tenocyte stem cells (Bi et al., 2007), we defined our cell population using FACS analysis and immunoblot for markers of stemness (Lui, 2015) (Supporting Information Data S2A) to exclude the possibility of our growth conditions causing tenocyte stem cell differentiation to a fibrochondrocyte phenotype. Supporting Information Data S2A demonstrates that human aged and young tenocytes have similar FACS profiles, both being positive for CD44, CD73, and CD90 and negative for stem cell markers CD19, CD34, CD45, and CD105. For both the aged and young human tenocyte populations, immunoblot of stem cell markers Oct‐4 and nucleostemin was negative (Supporting Information Data S2B). These findings indicate that the human cell populations were homogenous, composed of mature tenocytes and free of significant numbers of stem cells, furthering our observations that the altered phenotypic changes we observed were in mature tenocytes, not tenocyte stem cells.

### 2.3 Hypoxia regulates the aged tenocyte phenotype via modulating Rac1 Activity

**Figure 3:**
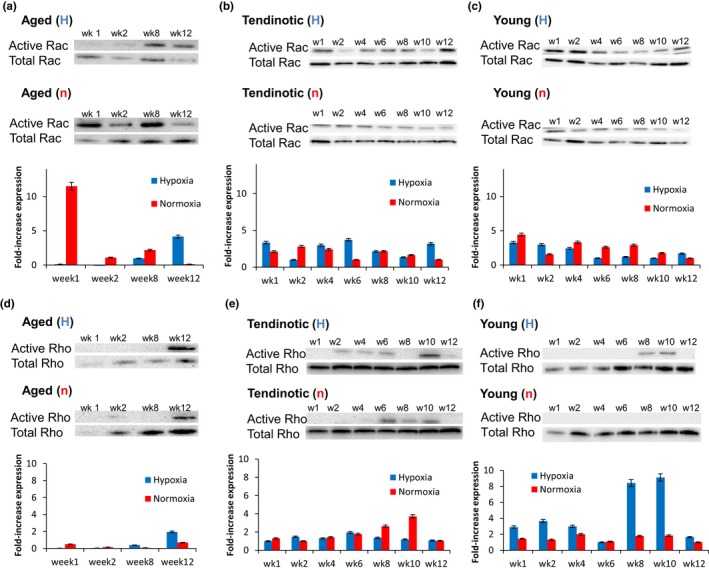
Hypoxia regulates the aged tenocyte phenotype via modulating Rac1 Activity. Human tenocytes from aged (a, d) young tendinotic (b, e) and young asymptomatic (c, f) patients were cultured at low density (aged at 1 × 10^3 ^cells/cm^2^; young tendinotic and young asymptomatic at 5 × 10^3 ^cells/cm^2^) in hypoxia (1% O_2_; (H)) or normoxia (21% O_2_; (n)) over twelve weeks and assayed for Rac1 (a, b, c) and RhoA (c, d, e) activity. Immunoblots were quantitated using ImageJ software. Error bars = *SEM*. *n* = 3. Representative Western blots as shown

Interested in the signaling mechanism responsible for the hypoxia‐driven phenotype changes observed in our tenocyte populations, we next asked whether changes in RhoA/Rac1 signaling underlied extracellular matrix production by aged, young tendinotic and young asymptomatic tenocytes in hypoxic (1% O_2_) and normoxic (21% O_2_) conditions. We observed stark differences between aged and young tenocytes in hypoxia at early timepoints, in that aged tenocytes displayed minimal Rac1 activation in hypoxia, but over 12‐fold increased Rac1 activation in normoxic conditions (21% O_2_) (Figure 3a). This hypoxia‐dependent decrease in Rac1 activity in aged tenocytes occurred concurrently with increased fibrochondrocyte and mineralized fibrochondrocyte molecular marker expression, collagen IIα1 and collagen X via Spearman's rho analysis (Figure 2m,o; Supporting Information Data S3). Also, this hypoxia‐dependent decrease in Rac1 activity in aged tenocytes appeared density‐dependent, as culture of aged tenocytes at high cell density in hypoxia resulted in increased Rac1 activity (Supporting Information Data S3A).

With respect to RhoA signaling, both aged and young tenocytes displayed low RhoA expression and activity in hypoxic and normoxic conditions at early timepoints (Figure 3d,f). In contrast, young tendinotic cells displayed increased RhoA activity in normoxic conditions compared to aged and young tenocytes (Figure 3e). Also, young hypoxic tenocytes displayed dramatically increased RhoA activity at later timepoints weeks 8 and 10 (Figure 3f) which correlated with decreased Rac1 activity (Figure 3c). This period of decreased Rac1 activity also correlated with increased fibrochondrocyte and mineralized fibrochondrocyte molecular marker expression, collagen IIα1 and collagen X (Figure 2m,o). Statistical analysis noted correlations between Rac1 activity and collagen IIα1 expression in aged and young tenocytes, as well as between Rho activity and collagen X expression at high cell densities (Supporting Information Data S4). Aged tenocytes, however, displayed increased RhoA activity at late timepoint week 12 (Figure 3d), independent of Rac1 activation and oxygen tension (Figure 3a).

These hypoxia‐dependent decreases in Rac1 activity, occurring concurrently with increased fibrochondrocyte and mineralized fibrochondrocyte markers, suggested that decreased Rac1 activity may be key to the tenocyte development of a fibrochondrocyte phenotype in aged cells. Furthermore, these results indicated that in young cells, decreased Rac1 activity concurrently with increased RhoA activity underlies tenocyte development of the fibrochondrocyte phenotype.

### 2.4 Rac1 inactivation modulates the aged tenocyte phenotype

**Figure 4:**
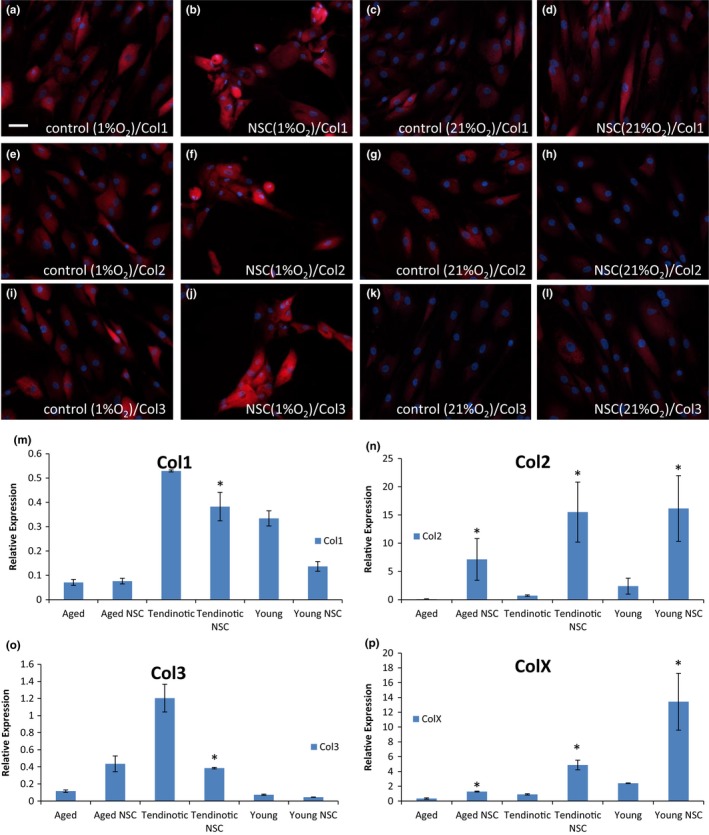
Rac1 inactivation modulates the aged tenocyte phenotype. Human tenocytes from aged patients were cultured in hypoxic (1% O_2_) or normoxic (21% O_2_) conditions in the presence or absence of Rac1 inhibitor NSC23766. Tenocytes were harvested after one week and stained for col1α2 (a–d), col2α1 (e–h) or col3 (i–l) expression via immunofluorescence. Bar = 50 μm. (m–p) Aged, young tendinotic, and young asymptomatic tenocytes were cultured in hypoxic (1% O_2_) or normoxic (21% O_2_) conditions in the presence (NSC) or absence (no drug) of Rac1 inhibitor NSC23766, harvested after one week and analyzed via qRT–PCR for col1α2 (m), col2α1 (n), col3 (o), or colX (p) expression. Error bars = *SEM*, *n* = 4. *p < 0.05

Given the early decreased Rac1 activation noted in hypoxia in aged tenocytes (Figure 3a) and late decreased Rac1 activation in young tenocytes (Figure 3c), both corresponding with increased fibrochondrocyte markers (Figure 2m,o), we next examined the effect of Rac1 inactivation on the aged, tendinotic, and young tenocyte phenotype using the Rac1 inhibitor, NSC23766 (Gao, Dickerson, Guo, Zheng, & Zheng, 2004). Aged, young tendinotic, and young asymptomatic tenocytes were plated at low cell density and cultured in hypoxia (1% O_2_) or normoxia (21% O_2_) in the presence (NSC) or absence (control) of NSC23766 and analyzed by immunofluorescence and qRT–PCR (Figure 4). Qualitatively, aged hypoxic tenocytes expressed more collagen IIα1 (Figure 4e) and collagen III (Figure 4i) than aged normoxic tenocytes (Figure 4g,k). When treated with NSC23766, aged hypoxic tenocytes displayed changes in cell shape, as seen by increased cell rounding, as well as increased expression of collagen IIα1 and collagen III (Figure 4f,j). Interestingly, these NSC‐dependent increases in collagen IIα1 and collagen III were not observed in aged normoxic tenocytes (Figure 4h,l), suggesting that the cue required for the development of a fibrochondrocyte phenotype is dependent on Rac1 inactivation in a hypoxic environment.

These effects of hypoxia on fibrochondrocyte marker expression in aged tenocytes treated with NSC23766 were quantitated via qRT–PCR and compared to the analogous normoxic condition (21% O_2_) (Figure 4m,n,o,p). We found that Rac1 inhibition in aged hypoxic tenocytes resulted in increased collagen IIα1 (Figure 4n, *p* < 0.05) and collagen X (Figure 4p, *p* < 0.05) as compared to the normoxic condition. When examining the effects of hypoxia on fibrochondrocyte marker expression in young tendinotic and asymptomatic tenocytes (Figure 4), we also found Rac1 inhibition resulted in large increases in collagen IIα1 (Figure 4n, *p* < 0.05) and collagen X (Figure 4p, *p* < 0.05) as compared to the normoxic condition. These findings suggest that the effects of Rac1 inhibition result in tenocyte development of a fibrochondrocyte phenotype regardless of cell type, in hypoxic conditions.

### 2.5 Differential, coordinated Rac1 and RhoA activity direct the human tenocyte phenotype

**Figure 5:**
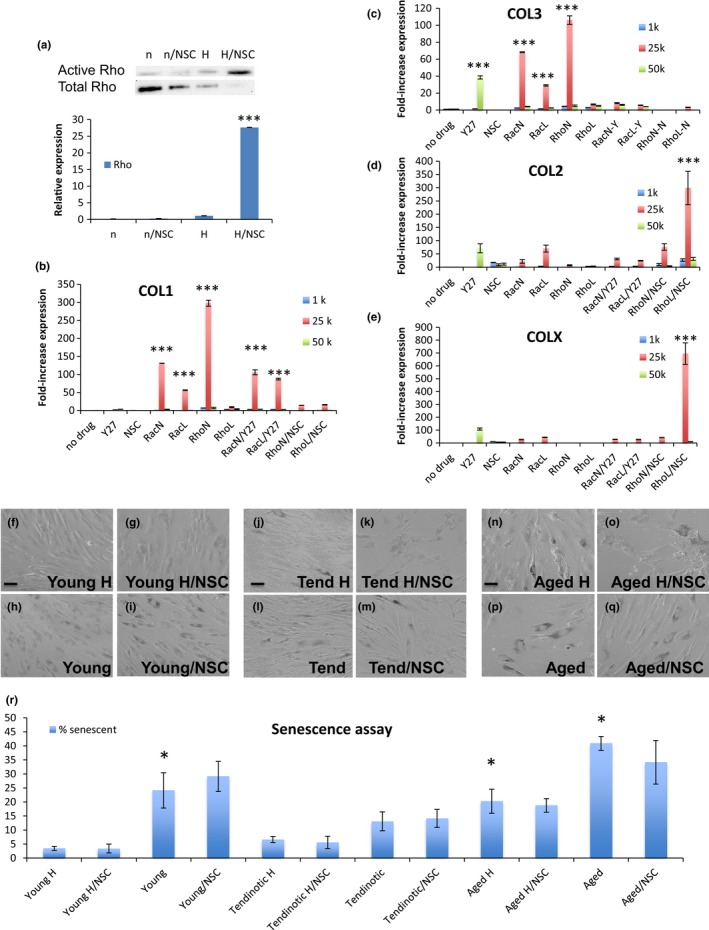
Differential, coordinated Rac1 and RhoA activity direct the human tenocyte phenotype. (a) Immunoblot of RhoA activity in young tenocytes at low density (5 × 10^3 ^cells/cm^2^) cultured in normoxic (n; 21% O_2_) or hypoxic (H; 1% O_2_) conditions, without or with Rac1 inhibitor NSC23766 (NSC) harvested after one week. Error bars = *SEM*, *n* = 4. *p < 0.05. Representative blot shown. (b–e) Young tenocytes cultured in normoxic conditions (21% O_2_) at low (5 × 10^3^cells/cm^2^), medium (25 × 10^3 ^cells/cm^2^), and high (50 × 10^3 ^cells/cm^2^) cell densities in the absence of drug (no drug), presence of ROCK inhibitor Y‐27632 (Y27), Rac1 inhibitor NSC23766 (NSC), dominant negative Rac1 (RacN), or RhoA (RhoN), constitutively active Rac1 (RacL), or RhoA (RhoL), without or with ROCK inhibitor Y‐27632 (/Y27) or Rac1 inhibitor NSC23766 (/NSC). Tenocytes were harvested after one week and analyzed for col1α2 (b), col3 (c), col2α1 (d), or colX (e) expression via qRT–PCR. Error bars = *SEM*, *n* = 4. ***p < 0.001. (f–q) Brightfield photomicrographs of young asymptomatic (“Young,” f–i) young tendinotic (“Tend,” j–m) or aged (“Aged,” n‐q) human tenocytes cultured in hypoxic (1% O_2_; (H)) or normoxic (21% O_2_) conditions in the presence (NSC) or absence of Rac1 inhibitor NSC23766, and harvested after one week for acid βgalactosidase senescence assay. Bar = 50 μm. (r) Quantitation of βgalactosidase‐positive staining cells as in (f–q) at time of harvest. Error bars = *SEM*, *n* = 4. *p < 0.05

Given the observation that Rac1 inhibition in hypoxia caused the fibrochondrocyte phenotype regardless of cell type (Figure 4m–p), but that RhoA activity differed in aged, young tendinotic versus young asymptomatic tenocytes (Figure 3d–f), we were interested in investigating the effect of hypoxia on RhoA activation in those cells treated with or without the Rac1 inhibitor (Figure 5a). In young asymptomatic cells, hypoxia resulted in minimal RhoA activation, which increased 30‐fold in the presence of concomitant Rac1 inhibition (Figure 5a). This result suggested that a role of hypoxia in tenocyte development of the fibrochondrocyte phenotype is in RhoA activation. This observation, coupled with the reciprocal pattern of Rac1/RhoA activation in young asymptomatic tenocytes in hypoxia, and loss of this pattern in aged and young tendinotic tenocytes (Figure 3), inspired the hypothesis that differential and coordinated Rac1/RhoA activity is necessary for tenocyte development of the fibrochondrocyte and mineralized fibrochondrocyte phenotype.

To investigate this hypothesis, we used constitutively active and dominant negative forms of Rac1 and RhoA GTPase in addition to pharmacologic inhibitors of Rac1 (NSC23766) and Rho‐kinase (ROCK, Y‐27632), to infect young normoxic (21% O_2_) tenocytes for one week prior to harvest for qRT–PCR analysis of tenocyte, fibrochondrocyte, and mineralized fibrochondrocyte markers. To observe whether there was an effect of cell density on the tenocyte phenotype, three densities (low 1k/cm^2^, medium 25k/cm^2^, and high 50k/cm^2^) of young tenocytes were used.

While there is a minimal effect of Rac1 inactivation alone on the young tenocyte phenotype in normoxic conditions (21% O_2_) (Figure 5b–e), we found that when young tenocytes expressed constitutively active RhoA concomitant with pharmacologic Rac1 inactivation, there was 300‐fold increased collagen IIα1, and 700‐fold increased collagen X expression (*p* < 0.001, Figure 5d,e). This effect was not observed in collagen III (Figure 5c). Interestingly, RhoA inactivity alone resulted in 300‐fold increase in collagen Iα2 and 100‐fold increase in collagen III (*p* < 0.001, Figure 5b,c). These findings were most significant in the medium cell density environment (25k/cm^2^) and indicate a strong role in young tenocytes for coordinated Rac1 inactivity coupled with RhoA activity as being necessary and sufficient for human tenocyte development of a fibrochondrocyte and mineralized fibrochondrocyte phenotype, irrespective of oxygen tension and patient age.

As senescence increases in aged cells and could be a potential confounding effect on our observations (Coppe et al., 2010), we cultured young, young tendinotic, and aged tenocytes in hypoxic (1% O_2_) and normoxic (21% O_2_) conditions, with or without the Rac1 inhibitor NSC23766, and harvested the cells for βgal senescence assay and p16 expression (Figure 5f–r, Supporting Information Data S5). Aged tenocytes demonstrate increased senescence compared to young asymptomatic tenocytes and young tendinotic tenocytes in normoxic as well as hypoxic conditions (*p* < 0.05, Figure 5f–r). Hypoxia decreased senescence in young asymptomatic and aged tenocytes (*p* < 0.05, Figure 5r). Immunoblot of p16 expression also demonstrated decreased p16 in young asymptomatic when compared to aged tenocytes, in hypoxic and normoxic conditions (Supporting Information Data S5). However, there was insignificant change in senescence and p16 expression in the presence of the Rac1 inhibitor NSC23766 (Figure 5, Supporting Information Data S5). These findings suggest that the effect of Rac1/RhoA activity on the human tenocyte phenotype is independent of senescence associated with p16 expression and acidic beta‐galactosidase activity.

### 2.6 Rac1 inhibition in human tendon explants recreates the aged diseased tendon phenotype

**Figure 6:**
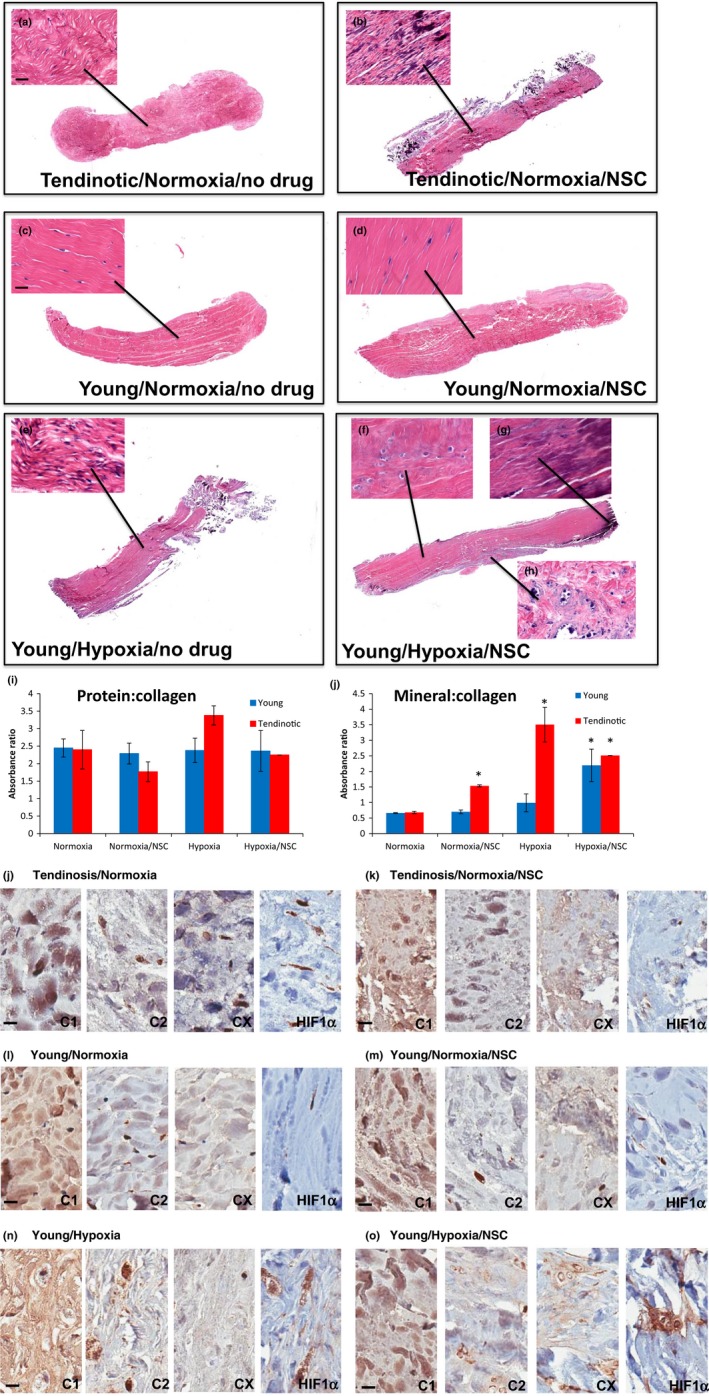
Rac1 inhibition in human tendon explants recreates the aged diseased tendon phenotype. Young diseased tendon (“Tendinotic,” a, b, j, k) or asymptomatic (“Young,” c–h, l–o) tendon explants cultured in normoxic conditions (21% O_2_; a, c, j, l), normoxic conditions with Rac1 inhibitor NSC23766 (NSC, b, d, k, m), hypoxia (1% O_2_; e, n), or hypoxia with NSC23766 (f, g, h, o) harvested after three months, stained for H&E (a–h) or via immunohistochemistry (j–o) for col1α2 (C1), col2α1 (C2), colX (CX), or HIF‐1α (HIF1α). Magnification of explants at 4x after routine H&E staining, with close‐up at 10x. Bar = 20 μm. *n* = 4. Representative explants shown. (i, j) FTIR of protein:collagen ratio (i) or mineral:collagen ratio (j) of explants as above. Error bars = *SEM*, *n* = 4. *p < 0.05

In order to investigate whether our hypothesis that Rac1 inhibition in hypoxia underlies tenocyte development of a fibrochondrocyte phenotype holds in a three‐dimensional tendon structure, we collected tendons from young affected patients (Tendinotic) undergoing tendon reconstruction, and young asymptomatic patients (Young) undergoing revision amputation for mutilating hand injury according to IRB‐approved protocols as previously, stripped the tendons of all surrounding tenosynovium, and cultured them as explants in normoxic (21% O_2_) or hypoxic (1% O_2_) conditions, in the absence or presence of Rac1 inhibitor NSC23766 (Figure 6a–j), without tension. In normoxic conditions, young tendinotic and young asymptomatic tendon explants displayed slight rounding of nuclei but no significant matrix metachromasia (Figure 6a,c), and expressed collagen 1α2 but not collagen 2α1 or collagen X (Figure 6j,l). However, when treated with NSC23766 in normoxic conditions, young tendinotic tendon explants displayed significant matrix metachromasia (Figure 6b) calcification (Figure 6b,j) and collagen X (Figure 6k), while asymptomatic tendon explants displayed minimal nuclear rounding and matrix metachromasia, with no calcification (Figure 6d,j). Interestingly, in hypoxia, young asymptomatic tendon explants displayed significant hypercellularity and mild nuclear rounding (Figure 6e), and when treated with NSC23766, there was prominent nuclear rounding and matrix metachromasia (Figure 6f), as well as a gradient of calcification at the edges of the tendon explant (Figure 6g) prominent calcification (Figure 6h,j) accompanied by expression of collagen 2α1, collagen X, and HIF1α (Figure 6o). FTIR revealed minimal effects of hypoxia and NSC inhibition on mature collagen ratios (Figure 6i), but significant increases in mineralization (Figure 6j). These findings suggest that Rac1 inhibition of human hypoxic tenocytes not subjected to tensile stress results in a fibrochondrocyte and mineralized fibrochondrocyte phenotype, even in a three‐dimensional environment.

## 3 DISCUSSION

**Figure 7:**
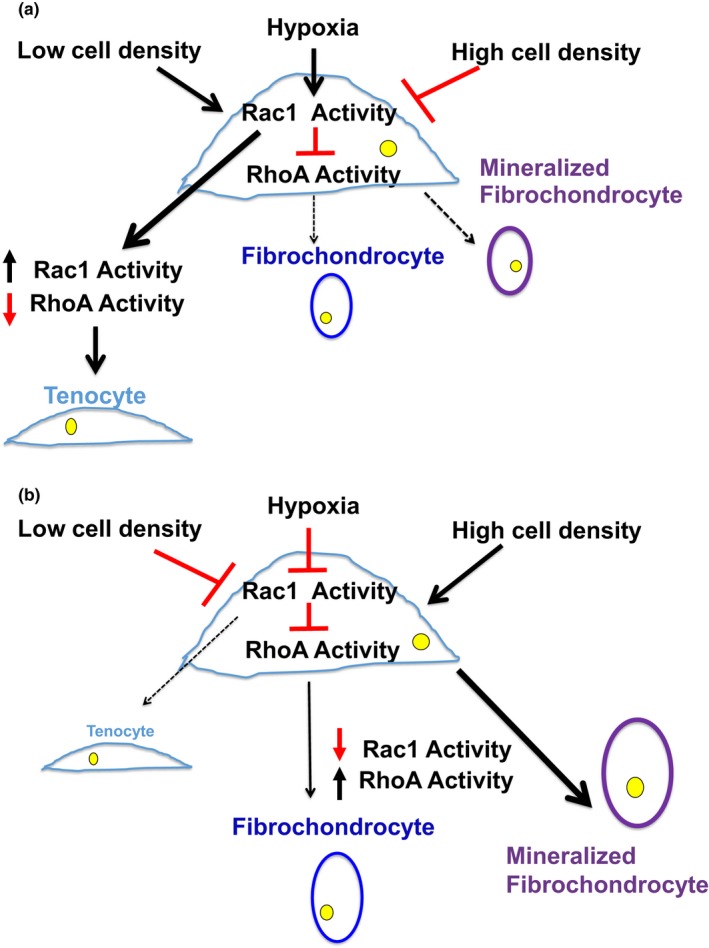
Model of age‐dependent tenocyte phenotype determination via hypoxic Rac1 inhibition. Microenvironmental cues including oxygen and cell density determine the human tenocyte phenotype. Hypoxia and low cell density in young tenocytes (a) activate Rac1 which inhibits RhoA, driving the tenocyte phenotype and repressing fibrochondrocyte and mineralized fibrochondrocyte formation. In aged tenocytes (b), hypoxia and low cell density inhibit Rac1 activity, allowing RhoA activation and formation of the fibrochondrocyte and mineralized fibrochondrocyte phenotype

The clinical entity of tendinosis is prevalent, debilitating, and age‐related. Correlations between the histopathologic phenotype of tendinosis—hyalinization and mineralization—and hypovascularity of tendons have been classically described in human tendinosis and aged tendon. The molecular mechanism responsible for the tenocyte phenotype changes in tendinosis, however, has heretofore been unknown. In this study, we investigated one possible mechanism of tendinosis development using immunohistochemical, biochemical, and molecular biology techniques on human patient samples and cells. We have discovered a novel relationship between hypoxia and inhibition of Rac1 GTPase activity at low cell density in aged tenocytes, which predisposes aged tenocytes to develop a fibrochondrocyte and mineralized fibrochondrocyte phenotype likely through RhoA disinhibition (Figure 7a,b). Furthermore, young tenocytes require concomitant Rac1 inhibition and RhoA activation to develop the fibrochondrocyte phenotype (Figure 7a), a finding observed exclusively in hypoxia. Given that tenocyte cell density affects Rac1/RhoA GTPase activity (Figure 3; Supporting Information Data S3, S3A and S4) and Rac1/RhoA activity mediates mechanical force transmission (Mui, Chen, & Assoian, 2016), we believe that the effect of tenocyte cell density on Rac1/RhoA activity as demonstrated here represents one way in which the tenocyte mechanical—in addition to oxygen—microenvironment effects tendinosis development.

Although the anatomic blood supply to tendons is well studied, the actual physiologic oxygen tension which supplies the tenocyte microenvironment is unclear. Established oxygen concentrations in mammalian tissues range from 1% to 7% in bone marrow and 1%–5% in interstitial tissues (Fehrer et al., 2007). In vitro studies of tenocytes cultured at 2%–7% O_2_ reveal decreased sensitivity of total protein and collagen production to hypoxia than other cell types, effects which are more pronounced at low cell density (Webster & Burry, 1982). Given the known effects of hypoxia and cell density on tenocyte phenotype maintenance (Yao et al., 2006; Zhang & Wang, 2018), the well‐documented decrease in vascularity of aged tendons (Rothman & Parke, 1965) and our own empiric studies, we chose to study human tenocytes isolated from aged and young individuals in hypoxic (1% O_2_) and normoxic (21% O_2_) conditions. In vitro tenocyte phenotype changes to fibrochondrocyte and mineralized fibrochondrocyte were dependent on hypoxia, and hypoxic tendon explants in which Rac1 was inhibited also demonstrated the fibrochondrocyte and mineralized fibrochondrocyte phenotype. Aged and young patient tendinosis samples as well as hypoxic tendon explant culture demonstrated increased HIF‐1α expression, which interestingly has been associated with Rac1 activity in other systems (Hirota & Semenza, 2001). Why hypoxia inhibits Rac1 activation in aged tenocytes at low cell density is unknown.

Interestingly, a similar pattern of tenocyte phenotype alteration—to fibrochondrocyte and mineralized fibrochondrocyte—occurs both at the embryonic and mature tendon‐bone insertion, termed the attachment unit and enthesis, respectively (Zelzer, Blitz, Killian, & Thomopoulos, 2015). In mice, the tendon‐bone attachment unit is formed from GDF5 progenitors co‐expressing Sox9 and Scx, regulated by the hedgehog (Hh) pathway (Dyment et al., 2015; Schwartz, Long, & Thomopoulos, 2015). Interestingly, decreased muscle loading increased the Hh‐responsive cell population, while ablation of the Hh‐responsive cells decreased fibrocartilage mineralization (Schwartz et al., 2015). The capacity for these progenitor cells to regenerate the mature enthesis was lost after terminal differentiation to fibrochondrocytes, however (Schwartz, Galatz, & Thomopoulos, 2017). While our studies were conducted using tenocytes isolated from mature human patients, the phenotypes we have observed in tendinosis are those that exist at the enthesis. Furthermore, given the relationships reported between Hh and the Rho/Rac family of GTPases, in particular the classic finding of increased Ihh signaling and ColX expression in Rac1‐deficient mice (Wang et al., 2007), we feel that our findings and model based on human patients and cells are symbiotic to those findings described in other developmental systems.

While we demonstrate a role for the Rho/Rac GTPases in tendinosis development, many other mechanisms affect the increased incidence of tendinosis with age. Aging results in increased matrix metalloproteinase activity in tenocytes (Yu et al., 2013) which causes decreased tendon strength (Dudhia et al., 2007). The mechanical contractile properties of aged tendon have been demonstrated to be age‐ and time‐dependent, thus affecting the mechanobiological environment of tenocytes (Lavagnino et al., 2014). Finally, the inflammatory component of tendinosis is incompletely understood, and dramatically changes with aging with respect to cytokine and prostaglandin expression (Ferrucci & Fabbri, 2018; Millar, Murrell, & McInnes, 2017). By correlating the roles of the oxygen and mechanical environment, we believe our findings enhance the current understanding of tendinosis development, and propose a molecular mechanism by which it occurs.

## 4 MATERIALS AND METHODS

### 4.1 Tendon preparation, surgical technique, and explant culture

Human tendon was isolated from patients undergoing revision amputation for traumatic hand injury using IRB‐approved protocols (#13D.238). Young asymptomatic tendon (“Young,” 19–42 y) and aged tendon (“Aged,” 65–81 years) were isolated from patients excluding workman's compensation, diabetes, pregnancy, systemic hormonal disorders or illness, revision surgery, and infection. Young affected tendon (“Tendinosis,” 35–44 years) was isolated from patients having had undergone repetitive stress injury presenting for surgical reconstruction. Multiple 3‐cm‐long areas of finger flexor tendon were harvested, dissected thoroughly from surrounding tenosynovium, and digested for tenocyte isolation or kept whole for explant culture. Aged tendinosis tissue was isolated from patients (75–80 years) undergoing A1 pulley release for flexor tendinosis.

### 4.2 Tenocyte isolation, cell culture, and FACS analysis

Human tenocytes were isolated from tendon tissue according to established protocols (Yao et al., 2006). In brief, tendon tissue was cut into 2 × 2 mm pieces and digested in collagenase solution (Worthington, collagenase type 2) in hg DMEM/Primocin (Invivogen), in 37°C incubator on shaker overnight. Digested tissue was filtered, centrifuged, and the cell pellet resuspended in growth media (hg DMEM, 10% pen/strep, L‐glutamine, primocin, 10% FBS (Gemini)) and plated at 5k cells/cm^2^. Tenocytes were maintained in normoxic (21% O_2_, ThermoForma Series II) or hypoxic (1% O_2_, Ruskinn Invivo2 hypoxia workstation) conditions. Cell culture media was changed weekly. Cells were passaged at 5k/cm^2^ after obtaining confluence unless described otherwise. Average time to confluence was 3 weeks. Experiments were performed using tenocytes of early passage (less than or equal to passage 4).

For FACS analysis, tenocytes were trypsinized and incubated with cell surface markers CD19, CD34, CD44, CD45, CD73, CD90, and CD105 according to manufacturer's instructions (BioLegend) and analyzed on FACS BD FACSCAlibur.

### 4.3 Immunohistochemistry, immunofluorescence, light microscopy stains, and FTIR

Human tendon surgical samples were fixed in 10% neutral buffered formalin and subjected to routine processing and H&E staining. For immunohistochemistry, 4‐um paraffin slides were deparaffinized in Shandon Varistain Gemini ES Autostainer. Antigen retrieval was performed with DAKO PTLink using Citrate Buffer (pH6.0) at 98˚C. Primary immunostaining was performed using antibodies against collagen 1α2 (gift from Dr. A. Fertala), collagen 2α1 (OriGene), collagen X (Abcam), and HIF‐1α (Abcam). Biotinylated anti‐rabbit or anti‐mouse (Vector Laboratories) secondary antibodies and ABC‐HRP complexes (Vector Laboratories) were applied. The signals were visualized using DAB substrate (DAKO) and counterstained with Hematoxylin.

For immunofluorescence, slides were incubated with primary antibodies: mouse monoclonal collagen 1α2, collagen 2α1, or collagen 3 (Abcam) at 4°C overnight. All slides were developed using goat anti‐mouse secondary antibody (Alexa Fluor) and DAPI to visualize the nuclei. Specimens were photographed using a fluorescence microscope (Eclipse E600, Nikon) equipped with a digital camera (DSQilMc, Nikon).

Alizarin Red staining was performed using kit 8678 (ScienCell) according to manufacturer's instructions, visualized using Nikon Eclipse TS100, and photographed using HTC One camera. Quantitation was performed according to manufacturer's instructions and quantitated using Tecan Infinite M1000 platereader. Alizarin red and alcian blue histopathologic stains were prepared and conducted according to Histopathology Core protocol.

β‐galactosidase assay (Millipore) was performed according to manufacturer's protocol.

FTIR was performed and analyzed according to standard protocols (Hanifi et al., 2013) using PerkinElmer FTIR Spectrometer Frontier/Spotlight 400. Human tendon surgical samples were fixed in 10% neutral buffered formalin and embedded in paraffin. Specimens were sectioned at 5 μm thickness on low emissivity slides (low‐e, Kevley Technologies, Chesterland, OH). Tendons were sectioned longitudinally, deparaffinized, and dehydrated prior to FTIR analysis. FTIR data were acquired from tendon sections at 8‐cm^−1 ^spectral resolution and 25‐µm spatial resolution with 32 scans per pixel. To calculate the relative amount of total protein to relative amount of mature collagen total protein in tendon, ratios of maximum absorbance at 1,640 cm^−1 ^(Amide I) to that at 1,338 cm^−1 ^(Amide III) were calculated. The same approach was undertaken to calculate relative amount of mineral deposited in the tendon to that of mature collagen. Ratios of maximum absorbance at 1,035 cm^−1 ^for mineral content to that at 1,338 cm^−1 ^(Amide III) were calculated.

### 4.4 Protein isolation and immunoblot

Tenocytes or tendon tissue was lysed in RIPA lysis buffer (Santa Cruz Biotech) and centrifuged; supernatant was loaded onto 12.5% SDS‐PAGE gels and transferred according to well‐established protocols. Antibodies to collagen Iα2 (rabbit polyclonal; gift of A. Fertala, Thomas Jefferson University), collagen IIα1 (Millipore), collagen III (Sigma), gapdh (Santa Cruz Biotech), RhoA (Santa Cruz Biotech), and Rac1 (Millipore) were used. Blots were imaged using ProteinSimple FluorChem E and quantitated using ImageJ software.

### 4.5 Quantitative RT–PCR

RNA was isolated using TRIZOL according to manufacturer's instructions (ThermoFisher Scientific). cDNA was prepared using 1 μg RNA and ecodry premix (Clontech). Quantitative RT–PCR was performed using Applied Biosystems StepOne qRT–PCR and primers for GAPDH, collagen Iα2, collagen IIα1, collagen 3, collagen X, scleraxis, and tenomodulin according to previously published primer sequences (Bertolo et al., 2012; Kyllonen et al., 2013; Wagenhauser et al., 2012). PowerUp SYBR Green (Applied Bioscience) was used as mastermix. All samples were run in duplicate. Data were prepared using ∆∆Ct and Microscoft Excel. Error reported is standard deviation from the mean.

### 4.6 Rho/Rac GTPase biochemical assay

Tenocytes were lysed in RIPA lysis buffer (Santa Cruz Biotech) and centrifuged, and supernatant added to PAK1‐GST beads for Rac1 activity assay (Millipore) or Rhotekin‐GST beads for RhoA activity assay (Millipore), and assays performed according to manufacturer's instructions. Assays were repeated in duplicate for each patient sample.

### 4.7 Pharmacologic and adenoviral agents

Rac1 inhibitor NSC23766 was used at 50 μM according to manufacturer's instructions and IC50 reported in the literature (Millipore) (Gao et al., 2004). ROCK inhibitor Y‐27632 was used at 10 μM according to manufacturer's instructions (Millipore). For adenoviral studies, constitutively active and dominant negative RhoA and Rac1 adenovirus were purchased, prepared, and titrated according to manufacturer's instructions (CellBioLabs).

### 4.8 Statistical analysis

qRT–PCR and Western blot quantitation data were analyzed using ∆∆CT (qRT–PCR), Spearman's rho and subsequent regression analysis as described (Maltenfort, 2015). qRT–PCR assays with two variables (drug, viral studies) were also assessed using ∆∆CT and Student's paired *t*‐test. Error bars depict *SEM*.

## CONFLICT OF INTEREST

None declared.

## AUTHOR CONTRIBUTIONS

R.M. involved in project idea, experimental design, data collection, data analysis, manuscript preparation, and review; R.W.E involved in data collection; B.J.O, A.S., involved in data collection and reviewed the manuscript; M.M performed the statistical analysis and reviewed the manuscript; S.P. and A.L.O reviewed the manuscript; I.M.S involved in project idea and reviewed the manuscript.
