# Supplementary material for: Tendinosis develops from age‐ and oxygen tension‐dependent modulation of Rac1 activity
Source: Aging Cell. 2019 Apr 2;18(3):e12934. doi: 10.1111/acel.12934 (PMC6516173; doi:10.1111/acel.12934)
Supplement: Supplementary file 1 [file ACEL-18-e12934-s001.pdf]

Gender	Age	Group	Mechanism of injury
M	25	Young	Sharp amputation; circular saw
M	35	Young	Sharp amputation; table saw
M	42	Young	Sharp amputation; straight blade
M	31	Young	Sharp amputation; table saw
F	30	Young	Sharp amputation; industrial
F	19	Young	Sharp amputation; industrial
F	35	Tendinotic	Repetitive use; delayed tendon
M	44	Tendinotic	Repetitive use; delayed tendon
F	42	Tendinotic	Repetitive use; delayed tendon
M	35	Tendinotic	Repetitive use; delayed tendon
F	81	Aged	Sharp amputation; vehicle edge
F	82	Aged	Sharp amputation; straight blade
M	75	Aged	Sharp amputation; straight blade
M	65	Aged	Sharp amputation; industrial
F	73	Aged tendinotic	Tendinopathy; pulley release
F	84	Aged tendinotic	Tendinopathy; pulley release
M	87	Aged tendinotic	Tendinopathy; pulley release
M	75	Aged tendinotic	Tendinopathy; pulley release
