# Supplementary material for: Tendinosis develops from age‐ and oxygen tension‐dependent modulation of Rac1 activity
Source: Aging Cell. 2019 Apr 2;18(3):e12934. doi: 10.1111/acel.12934 (PMC6516173; doi:10.1111/acel.12934)
Supplement: Supplementary file 2 [file ACEL-18-e12934-s002.pdf]

AGED						
	C1	C2	C3	CX	Tm	Scl
C1	1.00	0.31	0.79	-0.13	0.57	-0.03
C2	0.31	1.00	0.14	0.68	0.45	-0.20
C3	0.79	0.14	1.00	-0.25	0.81	-0.18
CX	-0.13	0.68	-0.25	1.00	0.16	-0.01
Tm	0.57	0.45	0.81	0.16	1.00	-0.45
Scl	-0.03	-0.20	-0.18	-0.01	-0.45	1.00

Supplementary Data 2

: Tendinotic						
: 5k						
	C1	C2	C3	CX	Tm	Scl
C1	1.00	-0.05	0.53	-0.01	-0.05	-0.04
C2	-0.05	1.00	-0.17	0.78	0.74	0.02
C3	0.53	-0.17	1.00	-0.45	-0.33	-0.48
CX	-0.01	0.78	-0.45	1.00	0.55	0.29
Tm	-0.05	0.74	-0.33	0.55	1.00	0.02
Scl	-0.04	0.02	-0.48	0.29	0.02	1.00

: Tendinotic						
: 25k						
	C1	C2	C3	CX	Tm	Scl
C1	1.00	-0.04	0.40	-0.29	-0.33	-0.33
C2	-0.04	1.00	-0.26	0.80	0.80	-0.42
C3	0.40	-0.26	1.00	-0.29	-0.43	-0.07
CX	-0.29	0.80	-0.29	1.00	0.83	-0.12
Tm	-0.33	0.80	-0.43	0.83	1.00	-0.10
Scl	-0.33	-0.42	-0.07	-0.12	-0.10	1.00

: Tendinotic						
: 50k						
	C1	C2	C3	CX	Tm	Scl
C1	1.00	-0.11	0.25	0.07	-0.03	-0.09
C2	-0.11	1.00	-0.41	0.91	0.93	0.47
C3	0.25	-0.41	1.00	-0.34	-0.40	-0.59
CX	0.07	0.91	-0.34	1.00	0.92	0.56
Tm	-0.03	0.93	-0.40	0.92	1.00	0.58
Scl	-0.09	0.47	-0.59	0.56	0.58	1.00

Supplementary Data 2

: Young						
: 5k						
	C1	C2	C3	CX	Tm	Scl
C1	1.00	0.47	0.51	-0.06	0.25	0.00
C2	0.47	1.00	0.35	0.46	0.68	0.24
C3	0.51	0.35	1.00	0.18	0.41	-0.11
CX	-0.06	0.46	0.18	1.00	0.57	0.76
Tm	0.25	0.68	0.41	0.57	1.00	0.43
Scl	0.00	0.24	-0.11	0.76	0.43	1.00

: Young						
: 25k						
	C1	C2	C3	CX	Tm	Scl
C1	1.00	0.30	0.74	-0.31	0.07	-0.18
C2	0.30	1.00	0.57	0.45	0.51	0.21
C3	0.74	0.57	1.00	-0.02	0.29	0.06
CX	-0.31	0.45	-0.02	1.00	0.41	0.56
Tm	0.07	0.51	0.29	0.41	1.00	0.55
Scl	-0.18	0.21	0.06	0.56	0.55	1.00

: Young						
: 50k						
	C1	C2	C3	CX	Tm	Scl
C1	1.00	0.10	0.68	0.02	0.04	-0.04
C2	0.10	1.00	0.19	0.40	0.81	0.39
C3	0.68	0.19	1.00	-0.34	0.02	-0.04
CX	0.02	0.40	-0.34	1.00	0.39	0.06
Tm	0.04	0.81	0.02	0.39	1.00	0.73
Scl	-0.04	0.39	-0.04	0.06	0.73	1.00

Supplementary Data 2
