# Supplementary material for: Tendinosis develops from age‐ and oxygen tension‐dependent modulation of Rac1 activity
Source: Aging Cell. 2019 Apr 2;18(3):e12934. doi: 10.1111/acel.12934 (PMC6516173; doi:10.1111/acel.12934)
Supplement: Supplementary file 3 [file ACEL-18-e12934-s003.pdf]

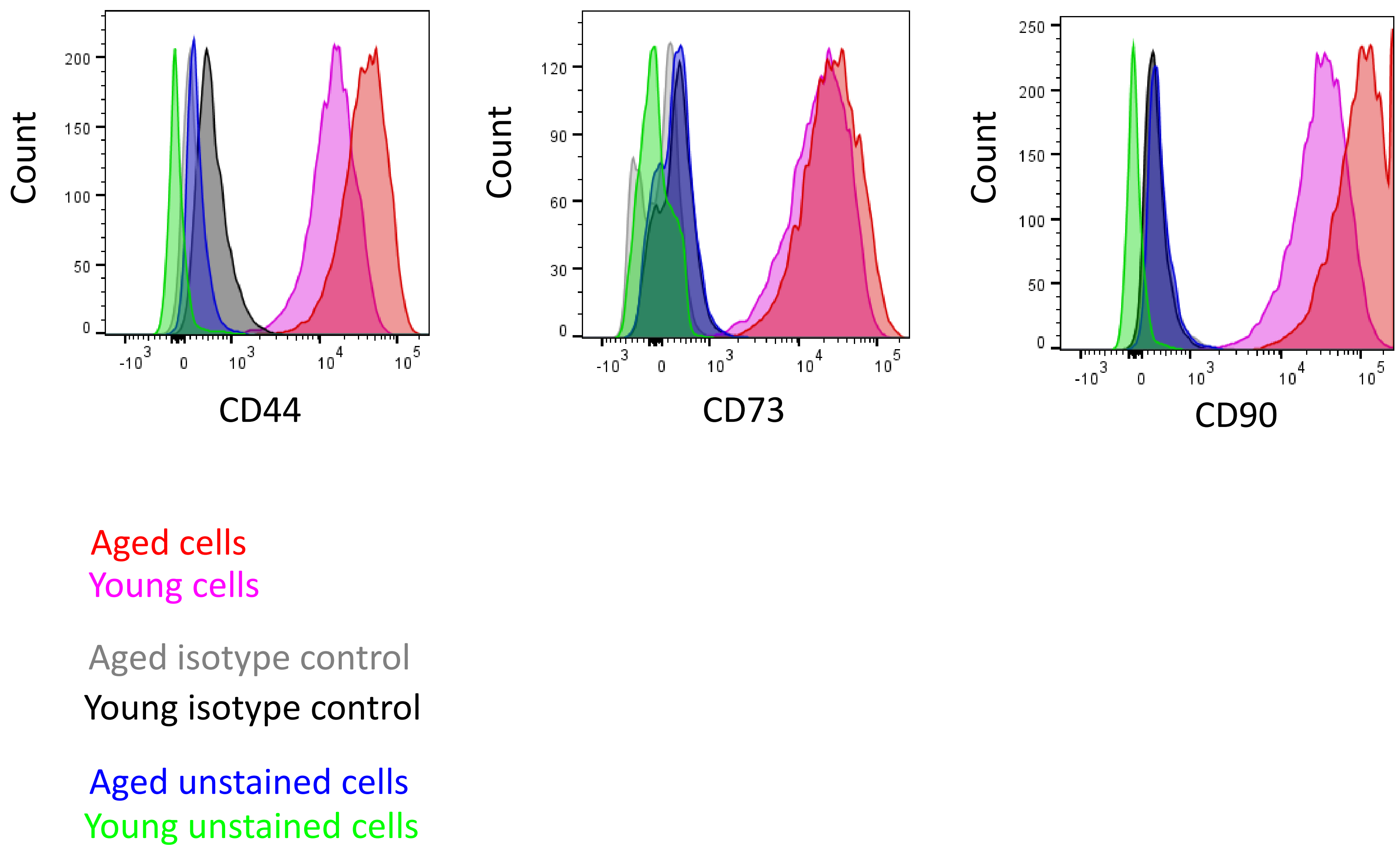

Supplementary Data 2a

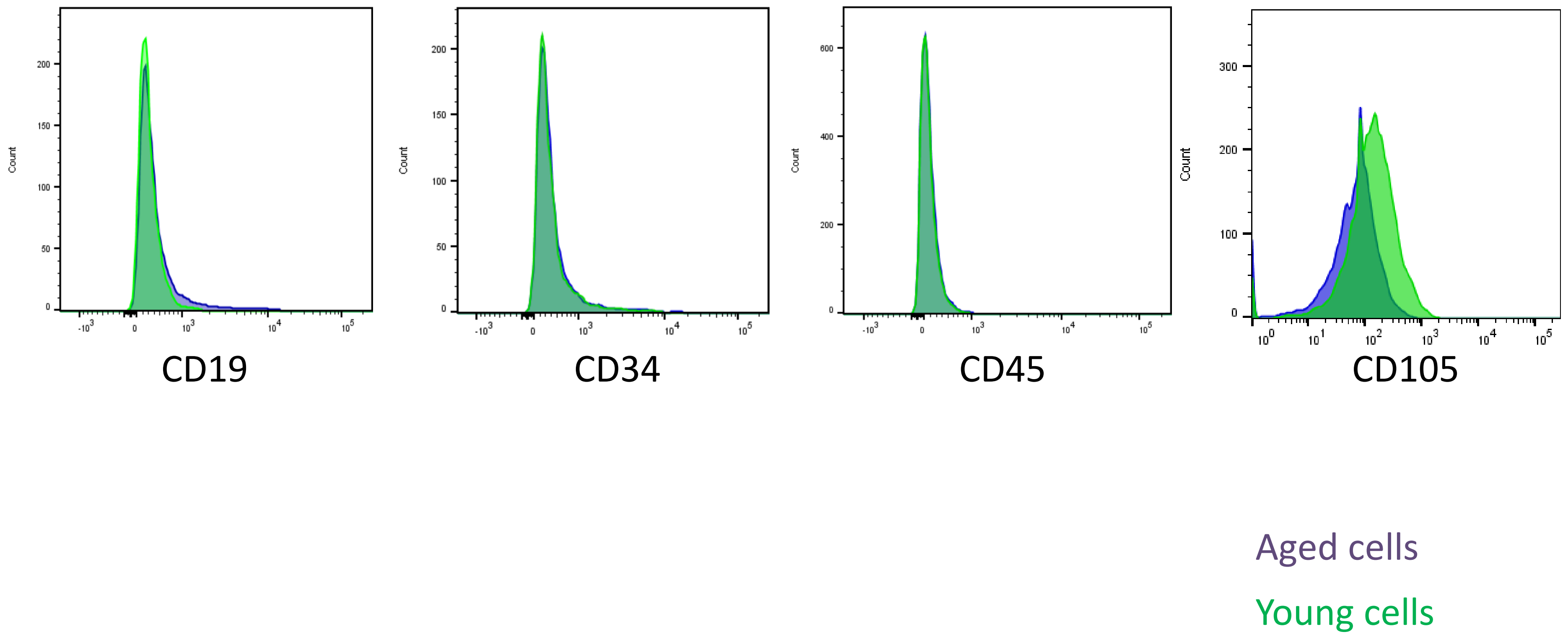

Supplementary Data 2a
