# Supplementary figures and images for: Tendinosis develops from age‐ and oxygen tension‐dependent modulation of Rac1 activity
Source: Aging Cell. 2019 Apr 2;18(3):e12934. doi: 10.1111/acel.12934 (PMC6516173; doi:10.1111/acel.12934)

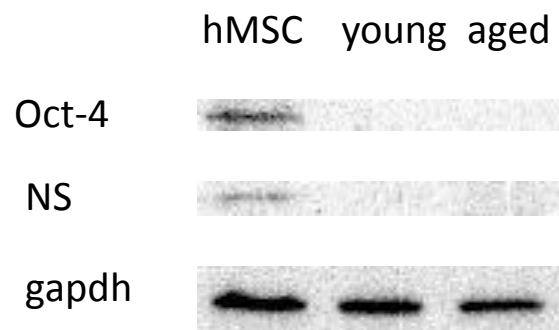

**Supplementary Data 2b**

Supplement: Supplementary file 4 [file ACEL-18-e12934-s004.pdf]

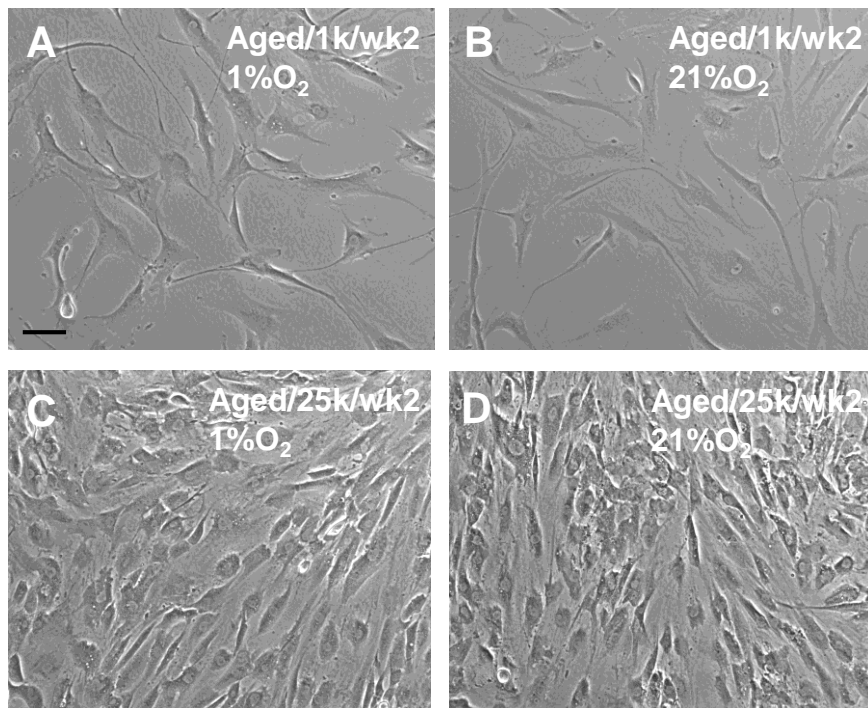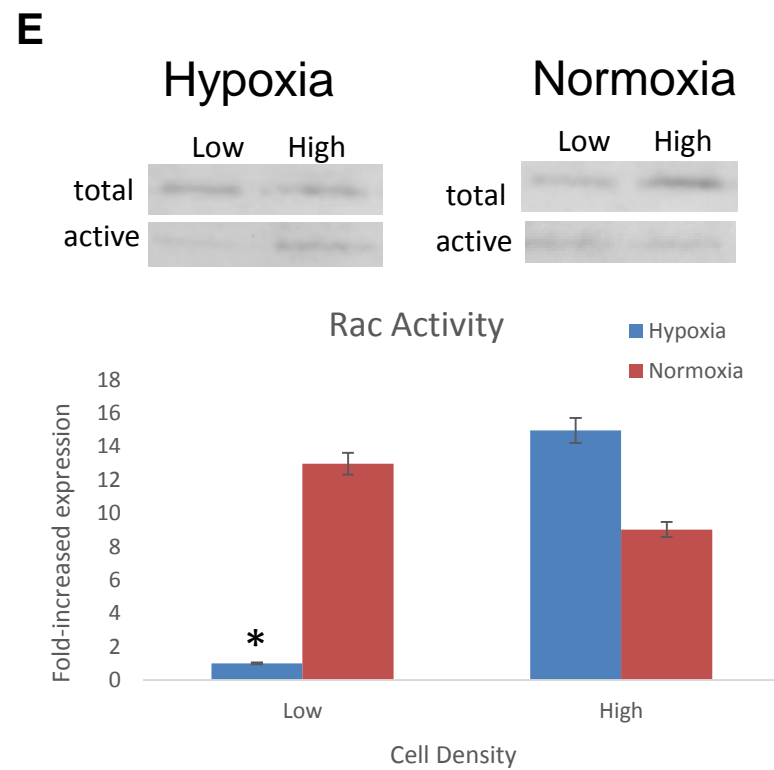

**Supplementary Data 3a**

Supplement: Supplementary file 6 [file ACEL-18-e12934-s006.pdf]

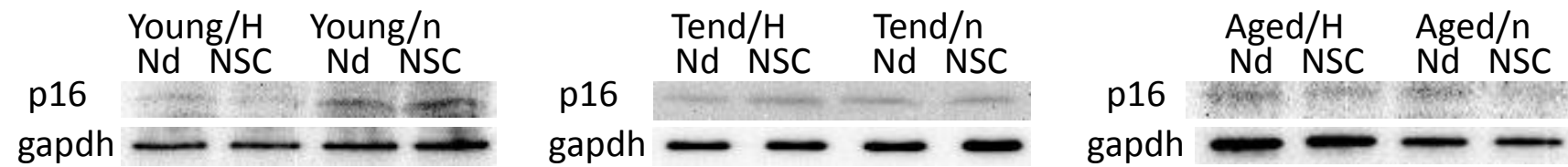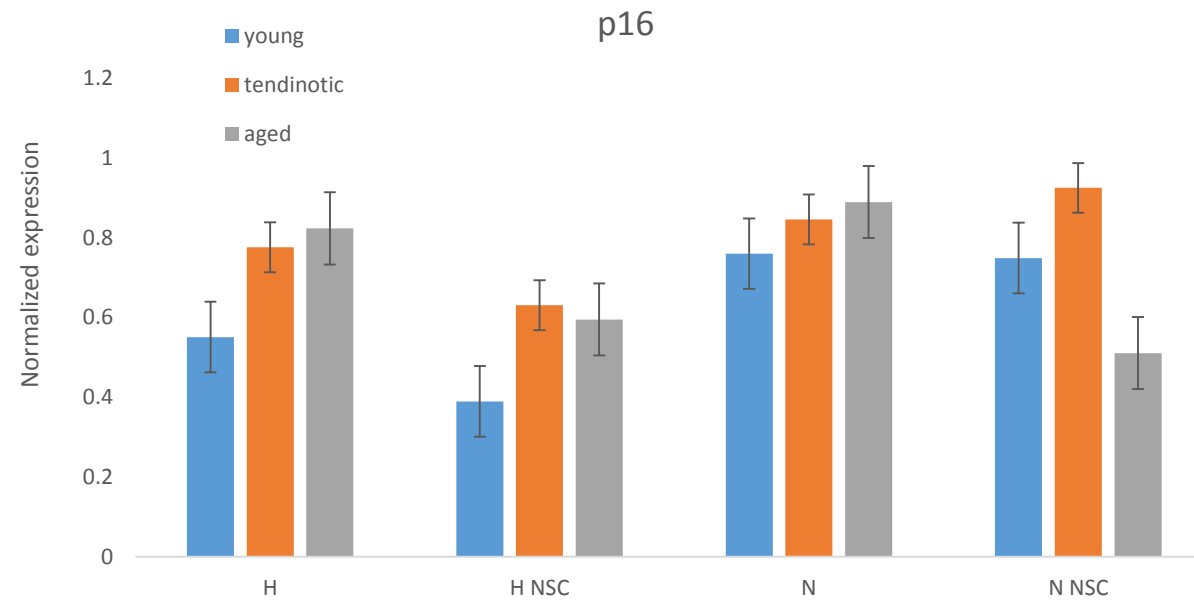

**Supplementary Data 5**

Supplement: Supplementary file 8 [file ACEL-18-e12934-s008.pdf]
