# Supplementary material for: Tendinosis develops from age‐ and oxygen tension‐dependent modulation of Rac1 activity
Source: Aging Cell. 2019 Apr 2;18(3):e12934. doi: 10.1111/acel.12934 (PMC6516173; doi:10.1111/acel.12934)
Supplement: Supplementary file 5 [file ACEL-18-e12934-s005.pdf]

		C1	C2	C3	CX	Tm	Rac	Rho
	C1	1.00	0.31	0.79	-0.13	0.57	-0.40	-0.20
AGED	C2	0.31	1.00	0.14	0.68	0.45	0.60	-0.80
	C3	0.79	0.14	1.00	-0.25	0.81	-0.80	0.40
	CX	-0.13	0.68	-0.25	1.00	0.16	0.40	0.00
	Tm	0.57	0.45	0.81	0.16	1.00	-0.40	0.20
	Rac	-0.40	0.60	-0.80	0.40	-0.40	1.00	-0.80
	Rho	-0.20	-0.80	0.40	0.00	0.20	-0.80	1.00
		C1	C2	C3	CX	Tm	Rac	Rho
TENDINOTIC	C1	1.00	-0.11	0.42	-0.09	-0.20	-0.30	0.19
	C2	-0.11	1.00	-0.28	0.84	0.90	0.13	0.14
	C3	0.42	-0.28	1.00	-0.37	-0.36	-0.16	-0.11
	CX	-0.09	0.84	-0.37	1.00	0.81	0.04	0.21
	Tm	-0.20	0.90	-0.36	0.81	1.00	0.15	0.15
	Rac	-0.30	0.13	-0.16	0.04	0.15	1.00	-0.03
	Rho	0.19	0.14	-0.11	0.21	0.15	-0.03	1.00
		C1	C2	C3	CX	Tm	Rac	Rho
	C1	1.00	0.32	0.62	-0.09	0.12	0.28	0.12
	C2	0.32	1.00	0.39	0.45	0.68	0.29	-0.04
	C3	0.62	0.39	1.00	-0.08	0.26	0.41	-0.21
YOUNG	CX	-0.09	0.45	-0.08	1.00	0.46	0.04	-0.01
	Tm	0.12	0.68	0.26	0.46	1.00	0.24	0.21
	Rac	0.28	0.29	0.41	0.04	0.24	1.00	-0.02
	Rho	0.12	-0.04	-0.21	-0.01	0.21	-0.02	1.00

Supplementary Data 3
