# Supplementary material for: Tendinosis develops from age‐ and oxygen tension‐dependent modulation of Rac1 activity
Source: Aging Cell. 2019 Apr 2;18(3):e12934. doi: 10.1111/acel.12934 (PMC6516173; doi:10.1111/acel.12934)
Supplement: Supplementary file 7 [file ACEL-18-e12934-s007.pdf]

: 5k							
: Young							
	C1	C2	C3	CX	Tm	Rac	Rho
C1	1.00	0.47	0.51	-0.06	0.25	0.28	-0.03
C2	0.47	1.00	0.35	0.46	0.68	0.65	0.08
C3	0.51	0.35	1.00	0.18	0.41	0.42	-0.16
CX	-0.06	0.46	0.18	1.00	0.57	0.32	-0.14
Tm	0.25	0.68	0.41	0.57	1.00	0.33	0.15
Rac	0.28	0.65	0.42	0.32	0.33	1.00	0.18
Rho	-0.03	0.08	-0.16	-0.14	0.15	0.18	1.00

DENSITY						
5k		C3	CX	Tm	Rac	Rho
	C3	1.00	0.03	0.13	0.22	0.10
	CX	0.03	1.00	0.40	0.23	0.30
	Tm	0.13	0.40	1.00	0.05	0.22
	Rac	0.22	0.23	0.05	1.00	-0.10
	Rho	0.10	0.30	0.22	-0.10	1.00
		C3	CX	Tm	Rac	Rho
	C3	1.00	0.14	0.03	0.41	0.20
25 k	CX	0.14	1.00	0.57	0.41	0.34
	Tm	0.03	0.57	1.00	0.33	0.09
	Rac	0.41	0.41	0.33	1.00	0.45
	Rho	0.20	0.34	0.09	0.45	1.00
50 k						
		C3	CX	Tm	Rac	Rho
	C3	1.00	0.04	-0.09	-0.20	0.20
	CX	0.04	1.00	0.61	-0.20	0.55
	Tm	-0.09	0.61	1.00	0.28	0.21
	Rac	-0.20	-0.20	0.28	1.00	-0.18
	Rho	0.20	0.55	0.21	-0.18	1.00

Supplementary Data 4
